# Large-scale demonstration of machine learning for the detection of volcanic deformation in Sentinel-1 satellite imagery

**DOI:** 10.1007/s00445-022-01608-x

**Published:** 2022-11-03

**Authors:** Juliet Biggs, Nantheera Anantrasirichai, Fabien Albino, Milan Lazecky, Yasser Maghsoudi

**Affiliations:** 1 COMET, School of Earth Sciences, University of Bristol, Bristol, UK; 2 Visual Information Laboratory, Department of Computer Sciences, University of Bristol, Bristol, UK; 3 University of Grenoble Alpes, ISTerre, Grenoble, France; 4 COMET, School of Earth and Environment, University of Leeds, Leeds, UK

**Keywords:** Satellite, Deformation, Global, Machine Learning, Volcano

## Abstract

Radar (SAR) satellites systematically acquire imagery that can be used for volcano monitoring, characterising magmatic systems and potentially forecasting eruptions on a global scale. However, exploiting the large dataset is limited by the need for manual inspection, meaning timely dissemination of information is challenging. Here we automatically process ~ 600,000 images of > 1000 volcanoes acquired by the Sentinel-1 satellite in a 5-year period (2015–2020) and use the dataset to demonstrate the applicability and limitations of machine learning for detecting deformation signals. Of the 16 volcanoes flagged most often, 5 experienced eruptions, 6 showed slow deformation, 2 had non-volcanic deformation and 3 had atmospheric artefacts. The detection threshold for the whole dataset is 5.9 cm, equivalent to a rate of 1.2 cm/year over the 5-year study period. We then use the large testing dataset to explore the effects of atmospheric conditions, land cover and signal characteristics on detectability and find that the performance of the machine learning algorithm is primarily limited by the quality of the available data, with poor coherence and slow signals being particularly challenging. The expanding dataset of systematically acquired, processed and flagged images will enable the quantitative analysis of volcanic monitoring signals on an unprecedented scale, but tailored processing will be needed for routine monitoring applications.

**Supplementary Information:**

The online version contains supplementary material available at 10.1007/s00445-022-01608-x.

## Introduction

Globally, over 800 million people live within 100 km of a volcano and the number of fatalities is increasing with time (Loughlin et al. 2015). By identifying precursory activity, volcano monitoring can provide forecasts of impending eruptions, reducing socio-economic impacts (Poland and Anderson 2020). Once adjusted for population growth, the vulnerability to volcanic hazards is actually falling as improved monitoring enables timely evacuations (Auker et al. 2013). However, the distribution of ground-based equipment remains unequal, and many low-middle income countries have little capacity, leaving a significant proportion of the ~ 1500 Holocene volcanoes essentially unmonitored, including many with high population exposure (Loughlin et al. 2015).

Satellite systems are well-suited for global environmental monitoring and measure a number of volcanic phenomena on a routine basis. In the last decade, there have been thousands of detections per year, most of which are co-eruptive thermal and gas emission signals. In contrast, many deformation signals are pre-eruptive (Furtney et al. 2018), thus offering the potential to inform eruption forecasting and crisis management (Biggs et al. 2014). Launched in 2014, the European Sentinel-1 constellation offers global coverage of radar images with a repeat cycle of 6 days at best, amounting to > 13 TB per day. The explosion in data represents a remarkable opportunity for global volcano monitoring, but has brought major challenges associated with manual inspection of imagery and timely dissemination of information, which can only be addressed with automated methods such as machine learning.

Proof-of-concept studies using convolutional neural networks (CNN) have demonstrated the potential of machine learning algorithms for detecting volcanic deformation in large InSAR datasets (Anantrasirichai et al. 2018). Following this, many new machine learning algorithms and architectures have been proposed for detecting and locating volcano deformation (Anantrasirichai et al. 2019a; 2019b; Bountos et al. 2022, 2021; Gaddes et al. 2019; Sun et al. 2020; Valade et al. 2019). However, these have only been tested on fairly small datasets (< 50,000 images) covering a limited range of volcano types, deformation characteristics and environmental conditions. For example, the dataset of Anantrasirichai et al. (2018) had two major limitations, despite being the largest available at the time. (1) Forty-five percent of the ~ 30,000 images were from Europe — where Sentinel-1 acquired images every 6 days — but this limited the range of conditions considered. (2) Only 4 of the ~ 900 volcanoes covered had deformation signals (Etna, Erte Ale, Sierra Negra and Cerro Negro), but this is not representative of the full range of volcano deformation characteristics (Biggs and Pritchard 2017; Ebmeier et al. 2018). Here we focus on the rapid expansion in the amount of available Sentinel-1 data in the last 4 years, from 30,000 images in 2019 to ~ 600,000 now. This new, expanded dataset provides an opportunity to test the existing algorithms on a wider range of deformation styles, atmospheric conditions and land cover, providing a benchmark against which future developments can be tested.

## Methods

**Fig. 1:**
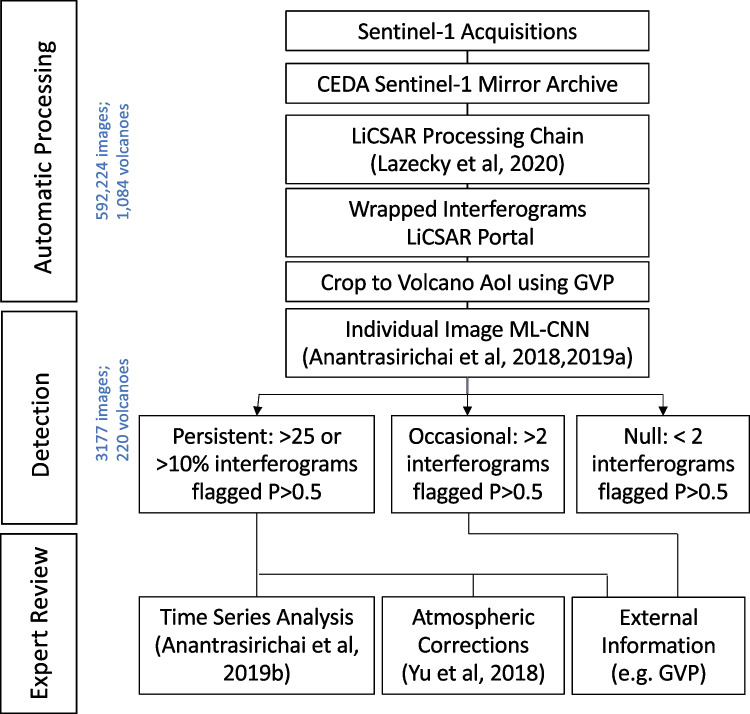
Flow chart summarising the methods used for the (1) automated processing of InSAR data, (2) detection of deformation and (3) expert review

We use a 3-stage approach to analyse the large dataset of satellite imagery available from Sentinel-1 (Fig. 1). The first step is the automatic generation of InSAR images using the COMET-LICSAR automated processing system (Lazecký et al. 2020). The next step is the automatic analysis of the processed images, and we use a deep learning approach to classify each image (Anantrasirichai et al. 2019b, 2018). For volcano monitoring, false negatives are far more problematic than false positive, so we use a conservative thresholding approach. Finally, we perform an expert review to identify the true positives and characterise the signal at each volcano making use of any external information available.

### Automated processing

Interferometric Synthetic Aperture Radar (InSAR) produces maps (‘interferograms’) of surface displacement in the satellite line of sight (los) by comparing the phase of the backscattered signal in successive SAR images. Here we use the COMET-LICSAR archive of processed Sentinel-1 radar images spanning November 2015–November 2020 (http://comet.nerc.ac.uk/COMET-LiCS-portal/). Sentinel-1 data is taken directly from the Centre for Environmental Data Analysis (CEDA) Sentinel Mirror Archive (SMA) and processed using the COMET-LICSAR automated processing system which is based on the GAMMA SAR processing software package and runs on the UK Centre for Environmental Data Analysis infrastructure JASMIN (Lazecký et al. 2020; Werner et al. 2000). The LiCSAR standard frames are a merge of 13 burst units for each of the 3 Sentinel-1 swaths, covering approximately 250 km by 250 km. The interferograms are geocoded onto a 0.001° × 0.001° grid using elevation data primarily from the Shuttle Radar Topography Mission (SRTM).

**Fig. 2:**
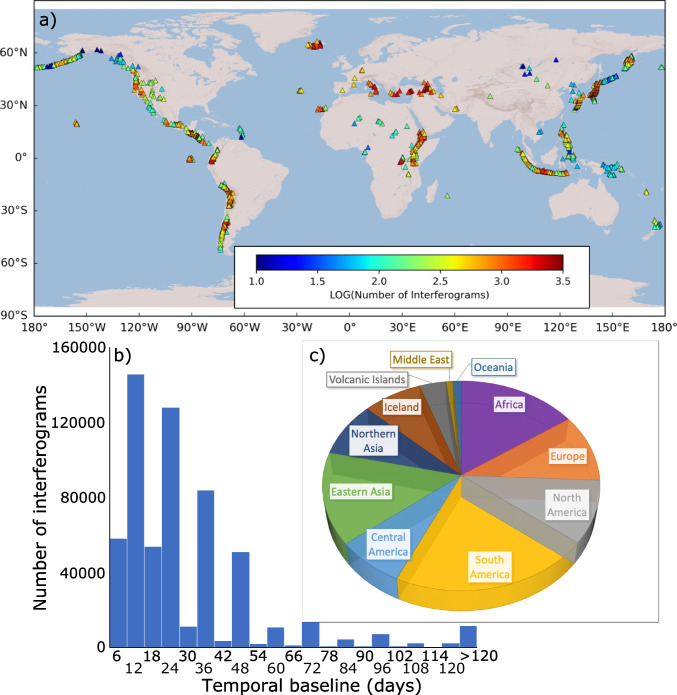
Distribution of testing dataset of ~ 600,000 images used in this study. (**a**) Worldmap showing the spatial distribution: colour dots indicate the number of Sentinel-1 interferograms calculated for each volcano. (**b**) Histogram showing the distribution of interferogram durations. (**c**) Pie chart showing distribution of images by region. Notice that the data are well-distributed and include a wide range of deformation styles, atmospheric conditions, durations and land cover types

The system generates differential interferograms connecting each Sentinel-1 acquisition to at least three successive acquisitions in time. The original Sentinel-1 mission consisted of two identical satellites, Sentinel-1A and Sentinel-1B, each of which has a revisit period of 12 days. Sentinel-1A was launched in 2014 and Sentinel-1B was launched in April 2016, but ceased operating in December 2021. In theory, this would generate interferograms of 6-, 12- and 18-day durations when both satellites are acquiring data and 12-, 24- and 36-day interferograms when just one satellite is acquiring data. However, the satellites do not always acquire data on every pass and the CEDA-SMA and LiCSAR archives are not yet complete. Although the majority of the inteferograms are shorter than 48 days in duration, missing acquisition means that longer duration interferograms are sometimes processed and > 10,000 interferograms are ≥ 120 days in duration (Fig. 2b). These long duration interferograms are particularly important for detecting slow deformation and assessing the effects of coherence on detectability.

We crop the area around each Holocene volcano to a 0.5° × 0.5° box centred on the coordinates provided by the Global Volcanism Programme (Global Volcanism Program 2013) generating 592,224 wrapped interferograms of 1084 volcanoes. In this dataset, ~ 10% of the interferograms are located over European volcanoes (compared to 43% in Anantrasirichai et al. (2018)) and there is good coverage of Latin American (28%) and Asian (22%) volcanoes, which are key volcanic settings (Fig. 2a,c). Unfortunately, some notable deformation events prior to or during the time period considered are not covered by the LiCSAR archive (e.g. Ambrym (Shreve et al. 2019), Semisopochnoi (DeGrandpre et al. 2019), Fogo (González et al. 2015)). However, we chose not to artificially bias the dataset by running additional processing at these volcanoes. Although incomplete, the dataset is an order of magnitude larger than that used by previous machine learning studies and allows us to explore a wider range of signal characteristics, volcanic processes, atmospheric conditions and land cover types.

### Detection

**Fig. 3:**
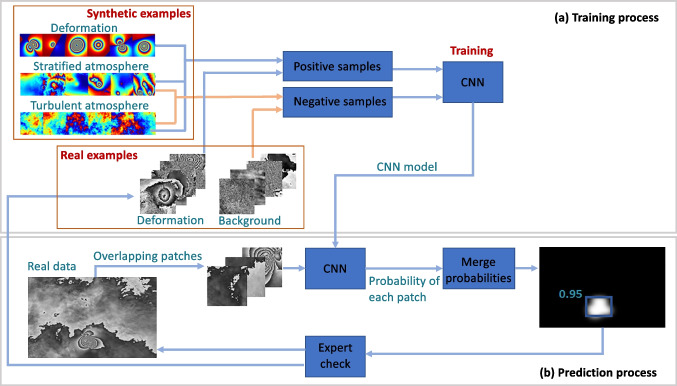
The machine learning framework developed by Anantrasirichai et al. (2018, 2019b). (**a**) During the training process, synthetic examples are used to train the CNN to obtain an initial model. The model is then retrained using real examples of both positive and negative results to improve the performance. (**b**) For the prediction process, the new interferograms are divided into patches, and the CNN outputs the probability of deformation in each patch. The probabilities are merged with Gaussian weights to produce a map and maximum probability for each image. Finally, the expert checks the results. Modified from Anantrasirichai et al. 2019b

Machine learning (ML), in particular, deep convolution neural networks (CNNs), is becoming increasingly popular for image classification tasks as they have proven to be efficient and are easy to adapt to many applications. However, to be successful, CNN models require a large training set of labelled data, balanced between classes. We apply the convolution neural network (CNN) proposed by Anantrasirichai et al. (2018), which was designed to distinguish between volcano deformation and atmospheric artefacts in wrapped interferograms. This approach fine-tunes the AlexNet architecture (Krizhevsky et al. 2012) using a combination of real (Anantrasirichai et al. 2018) and synthetic (Anantrasirichai et al. 2019b) training data (Fig. 3). The wrapped interferogram is converted to 8-bit (grayscale) data and divided into overlapping patches of 224 × 224 pixels, which are repeatedly shifted by 28 pixels to cover the image.

For this study, we use the 2-class model of Anantrasirichai et al. (2019b), which is trained to distinguish interferograms that consist of both deformation and atmospheric signals from those with just atmospheric signals (“D + S + T vs S + T” where D, deformation signal; S, stratified atmospheric signal; T, turbulent atmospheric signal). The probabilities of deformation in each patch are merged, and the images are flagged as containing deformation when the maximum probability, P, is greater than 0.5 (Anantrasirichai et al. 2018).

### Expert review

Finally, we use a range of tools to inspect the flagged interferograms and categorise the signals according to the processes responsible. This allows us to identify false positives and investigate the range of mechanisms represented and the associated detection thresholds.

We class the volcanoes as ‘persistent detections’ if they have either > 25 detections or > 10% of detections per total number of interferograms, and ‘occasional detections’ if 2–25 inteferograms were flagged. Since the LiCSAR system automatically connects each SAR acquisition to at least three successive acquisitions in time, any rapid transient deformation should appear in at least 6 interferograms. Here we use a conservative threshold of two detections to allow for incoherence, processing errors etc. For volcanoes with persistent detections, we use time-series and atmospheric correction approaches to investigate the characteristics of the signal, while volcanoes with occasional detections are checked visually.

To form time series for volcanoes with persistent detections, we apply the method used by Albino and Biggs (2021): the interferograms are unwrapped using snaphu (Chen and Zebker 2000; Lazecký et al. 2020) and a least-squares approach is used to retrieve cumulative displacement maps for each time-step (Schmidt and Burgmann 2003). Each time-step is re-wrapped to produce fringes and the CNN algorithm is applied. The choice of wrapping interval is arbitrary and Anantrasirichai et al. (2019a) showed that small intervals produce many false positives while large intervals are not able to detect slow deformation. Thus, we compute the final probability by averaging the probability at wrap intervals of 2.8 cm, 1.4 cm, 0.7 cm and 0.35 cm (Anantrasirichai et al. 2019a). For signals dominated by a seasonal component, we apply automatically generated atmospheric corrections using the COMET-GACOS system (Yu et al. 2018) which is based on the operational HRES weather model produced by the European Centre for Medium-Range Weather Forecasts (ECMWF) data (0.1° grid, 137 vertical levels, and 6-h intervals).

## Results

**Fig. 4:**
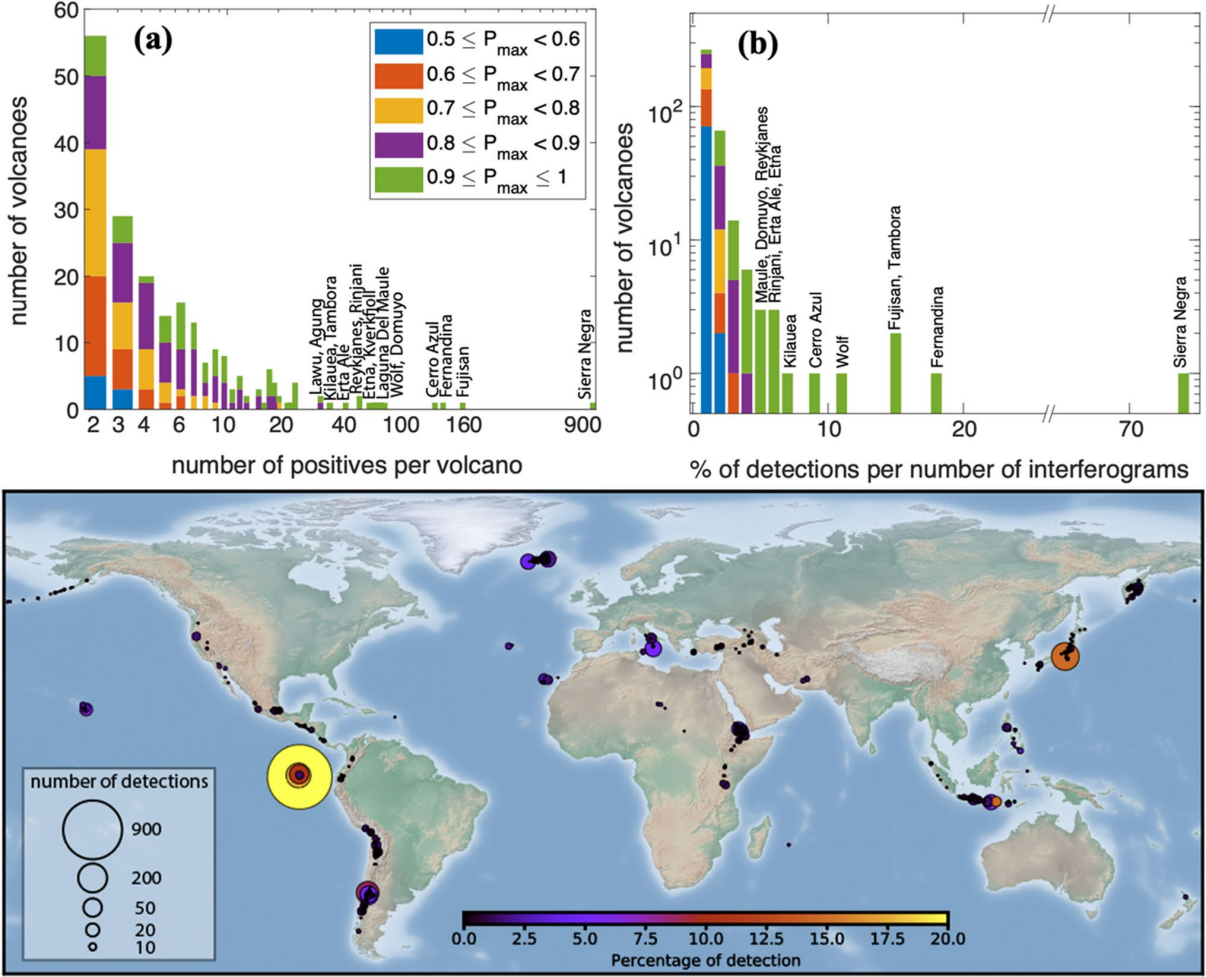
Outputs of the automated processing and machine learning detection of deformation organised by volcano. (**a**) Number of detections (*P* > 0.5) per volcano (log-scale), coloured by maximum probability, *P*_max_. (**b**) Percentage of detections (*P* > 0.5) per volcano (log-scale), coloured by maximum probability, *P*_max_. (**c**) Spatial distribution of detections (*P* > 0.5) from Table 1. Size represents the number of detections and colour the percentage of detections. Further details of volcanoes with a high number or percentage of detection are provided in Table 1 and the ‘Results’ section

The algorithm flagged 3323 wrapped interferograms of 366 volcanoes (Fig. 4). Of these, 146 volcanoes were only flagged once. Re-classifying volcanoes detected only once leaves 3177 interferograms flagged at 220 volcanoes. Volcanoes with a small number or proportion of detections tended to have lower maximum probabilities (0.5 < *P* < 0.9), while volcanoes with large numbers (> 20) or a high percentage (> 5%) of detections have very high maximum probabilities (*P* > 0.9). In this section, we first describe the 16 volcanoes with persistent detections in detail, and then highlight interesting examples from the 215 volcanoes with occasional detections.

### Persistent detections

#### The Galapagos Islands

**Fig. 5:**
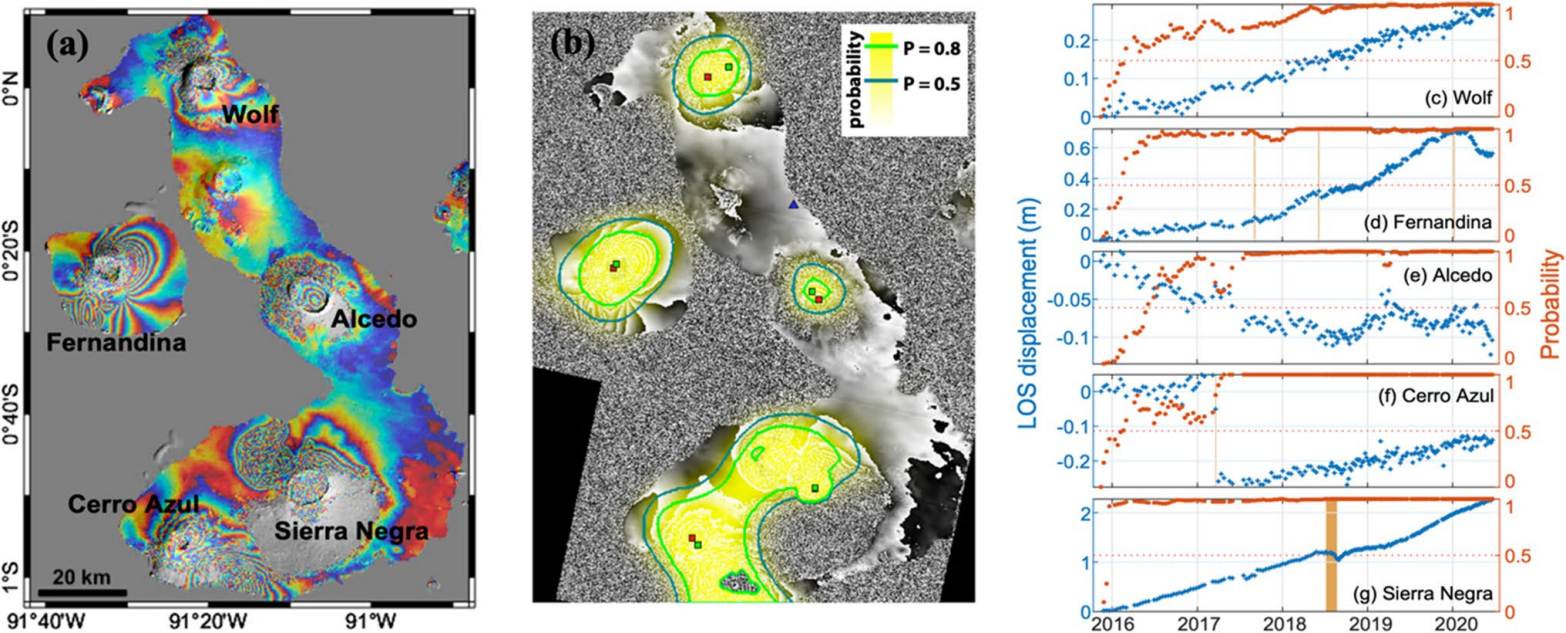
Automated InSAR processing and detection at Galapagos volcanoes. (**a**) Cumulative displacement between November 2015 and November 2020 displayed as wrapped fringes, where each fringe represents 2.8 cm of displacement in the line of sight, (**b**) detection probability output by the Convolutional Neural Network. Red square: volcano summit (Global Volcanism Program 2013); green square: selected point; blue: reference point. (**c**–**g**) timeseries showing displacement (blue) and probability (red) at (**c**) Wolf, (**d**) Fernandina, (**e**) Alcedo, (**f**) Cerro Azul and (**g**) Sierra Negra. Eruption events are marked in orange in (**c**–**g**)

Of the 3323 detections, 1247 (38%) were from volcanoes in the Galapagos Islands, with 904 from Sierra Negra alone (Fig. 4, Table 1). Sierra Negra erupted in 2005 and 2018. The 2018 eruption caused 8.5 m of subsidence (Bell et al. 2021), and the CNN flagged all individual wrapped interferograms spanning the 2018 eruption at *P* > 0.80. Magma recharge between eruptions caused continuous, but not constant, deformation. The total uplift of 6.5 m between 2005 and 2018 (Bell et al. 2021) makes this one of the fastest and longest-lived deformation episodes ever recorded (Biggs and Pritchard 2017), and an easy target for automated detection algorithms (Gaddes et al. 2019). Over 80% of the individual wrapped interferograms were flagged at *P* > 0.9 (Table 1). However, the extreme co-eruption signal was not accurately unwrapped by the automated processing system, aliasing the time series and such that only ~ 2 m of deformation was recorded in the satellite line of sight (Fig. 5g). Nonetheless, the cumulative time series reached the detection threshold (*P* > 0.5) within 30 days and was not sensitive to any further changes in deformation rate (Fig. 5g).

**Table 1:** Machine learning classification of automatically processed Sentinel-1 images for selected volcanoes between November 2015 and November 2020

Volcano name	Number images	Probability						% detections
		> 0.9	0.8–0.9	0.7–0.8	0.6–0.7	0.5–0.6	Sum	
Sierra Negra, Galapagos	1225	771	47	35	29	22	904	73.8
Fujisan, Japan	1127	73	34	21	17	20	165	14.6
Fernandina, Galapagos	742	34	20	13	32	32	131	17.7
Cerro Azul, Galapagos	1225	35	18	21	30	15	119	8.9
Wolf, Galapagos	742	5	18	20	24	14	81	10.9
Domuyo, Argentina	1399	14	15	7	15	12	63	4.5
Laguna Del Maule, Chile	1370	28	13	9	6	5	61	4.5
Etna, Italy	1149	13	7	11	11	16	58	5.1
Kverkfjoll, Iceland	2537	11	12	8	5	19	55	2.2
Krýsuvík-Trölladyngja Volcanic Field, Iceland	1174	37	6	3	1	2	49	4.2
Rinjani, Indonesia	890	10	19	7	3	10	49	5.5
Erta Ale, Ethiopia	798	9	18	8	2	5	42	5.3
Kilauea, Hawaii	489	26	6	1	1	0	34	7.0
Tambora, Indonesia	176	6	8	7	4	8	33	18.8
Agung, Indonesia	890	2	1	9	12	7	31	3.5
Lawu, Indonesia	1847	1	6	6	8	10	31	1.7

Three other Galapagos volcanoes were also flagged more than 80 times: Wolf, Cerro Azul and Fernandina (Fig. 5a). Between 2016 and 2020, both Wolf and Fernandina were continuously deforming, with the time series reaching *P* > 0.5 after just 3.2 and 4.3 months respectively (Fig. 5c,d). At Wolf, the uplift rate of 6 los cm/year was significantly faster than the rate of 3 los cm/year observed prior to the 2015 eruption (Xu et al. 2016). Uplift at Fernandina continued between November 2015 and January 2020, despite minor effusive eruptions in September 2017 and June 2018 (Vasconez et al. 2018), but a similar small eruption in January 2020 caused a reversal in deformation, and was followed by subsidence at a rate of 28 los cm/year. Cerro Azul experienced a non-eruptive deformation event in March 2017 attributed to the intrusion a sill at a depth of 5–6 km (Guo et al. 2019) (Fig. 5f). Although there was little deformation prior to this intrusion, the proximity to the large deformation signal at Fernandina caused the machine learning algorithm to assign a background probability of 0.5–0.8 (false positive), which increased to > 0.95 (true positive) after the intrusion.

#### Eruptions elsewhere

**Fig. 6:**
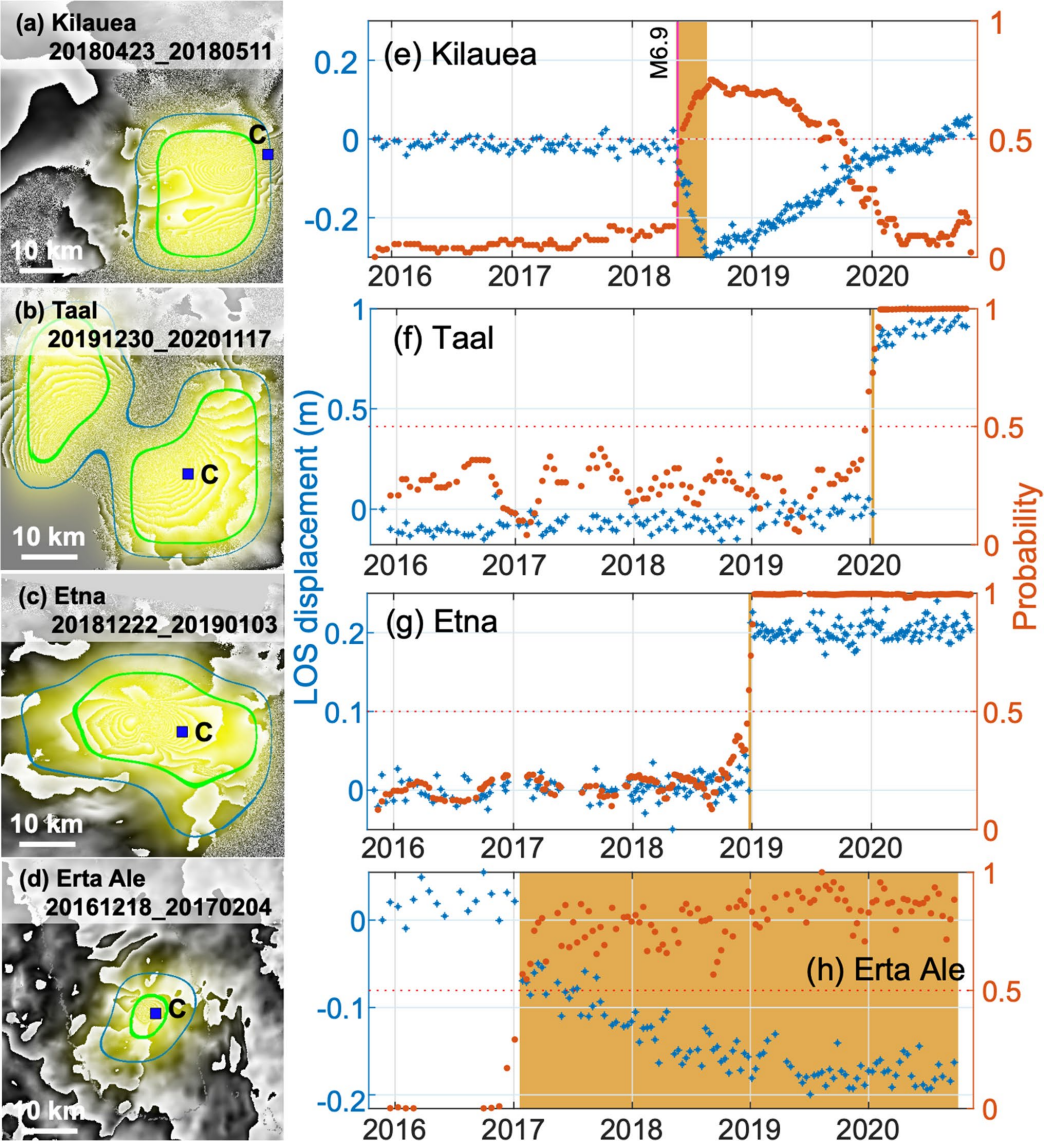
Automated InSAR processing and detection of major eruptions. (**a**–**d**) Individual wrapped interferograms with CNN probabilities super-imposed. Each fringe represents 2.8 cm of displacement in the satellite line of sight, and the semi-transparent yellow overlay represents the probability from the CNN, with contours at *P* = 0.5 and *P* = 0.8 (see Fig. 4b for scale). The blue squares labelled ‘C’ show the points selected for time-series analysis in e–h. (**e**–**h**) Cumulative displacement time series of unwrapped interferograms (blue) with associated probabilities (red). (**a**, **e**) Kilauea, USA; (**b**, **f**) Taal, Philippines; (**c**, **g**) Etna, Italy; (**d**, **h**) Erta Ale, Ethiopia. Orange colours denote eruptive periods

The CNN detected ground deformation associated with eruptions at the following: Kilauea, Hawaii, in 2018 (Neal et al. 2019); Erta Ale, Ethiopia, in January 2017 (Moore et al. 2019); and Etna, Italy, in December 2018 (De Novellis et al. 2019) (Fig. 4). The deformation at Kilauea was large magnitude and complex in both space and time (Neal et al. 2019) and the CNN flagged deformation in 35 individual interferograms at Kilauea (Fig. 4, Fig. 5a). The time series probability reaches *P* > 0.5 on 4 June 2018 (Fig. 6e), 1 month after the eruptive fissures first opened (Neal et al. 2019). Following the end of the eruption, the sign of the deformation reversed and the probability associated with the cumulative deformation dropped below the detection threshold on 3 October 2019. This illustrates the challenges associated with using cumulative time series to detect changes in rate (Supplementary Fig. 1 in the online resource).

**Fig. 7:**
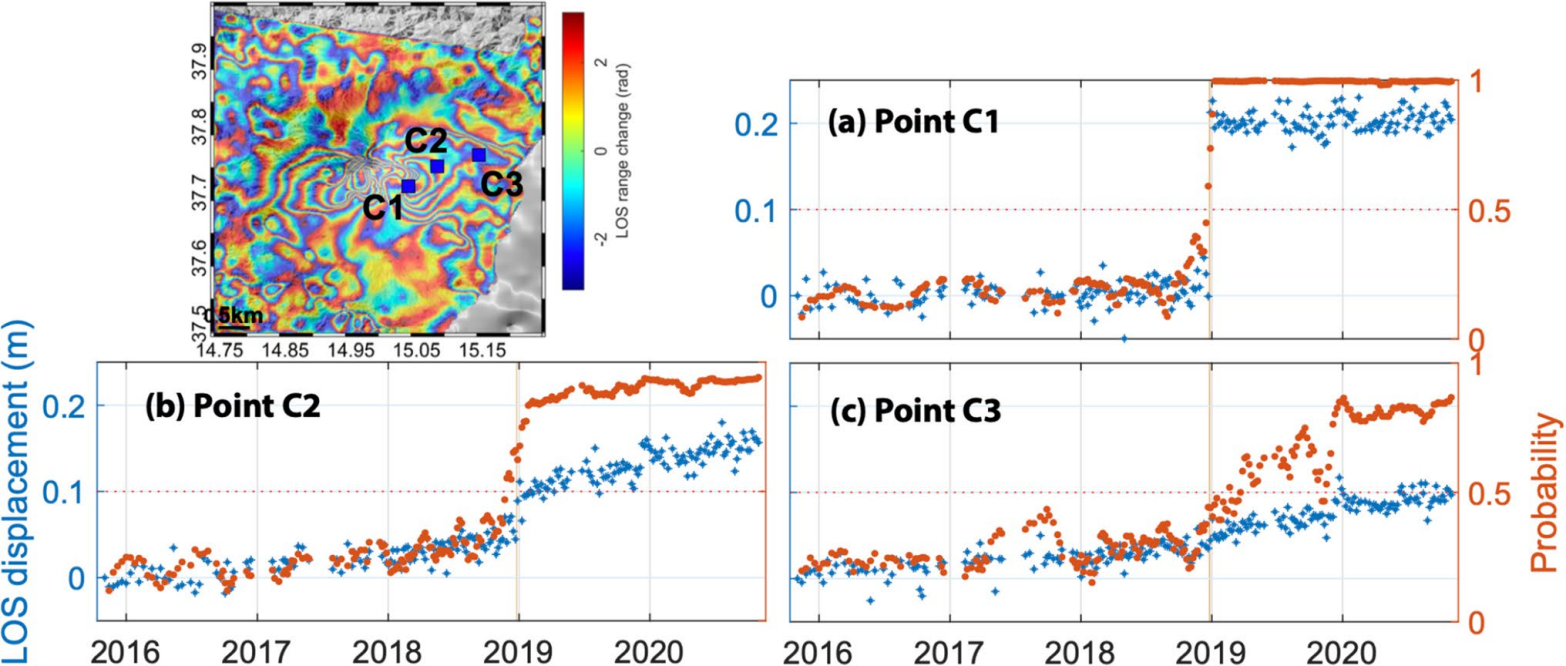
Automated processing and machine learning results for Etna, Italy. Cumulative displacement map (wrapped modulo 2.8 cm) for November 2015–November 2020. (**a**–**c**) Timeseries showing cumulative displacement (blue) and probability (red) for selected points. (**a**) Point C1 is close to the summit and deformation is dominated by the dyke intrusion. (**b**) Point C2 is located high on the flank and shows a combination of displacement caused by the intrusion and flank sliding. (**c**) Point C3 is located low on the flank and displacement is dominated by the slow flank sliding. Eruption dates are shown in orange

Fissure eruptions at Erta Ale, Ethiopia, in January 2017 (Moore et al. 2019) and Etna, Italy, in December 2018 (De Novellis et al. 2019) are flagged in more than 40 wrapped interferograms each (Fig. 4, Table 1). These eruptions were associated with small dyke intrusions which generate a characteristic two-lobed deformation pattern (Fig. 6c,d). At pixels close to the intrusions, the time-series are dominated by the intrusive process (Fig. 6g,h), but more detailed analysis shows additional long-term deformation signals, associated with flank sliding at Etna and the shallow magma system at Erta Ale (Supplementary Fig. 2 in the Online Resource). These patterns are spatially distinct and can be separated by selecting time series from different pixels. For example, Fig. 7 shows 3 selected pixels at Etna: C1 is close to the summit and dominated by the dyke intrusion, C3 is low on the flank and dominated by flank sliding and C2 is a combination of both signals.

#### Systems experiencing volcanic unrest

**Fig. 8:**
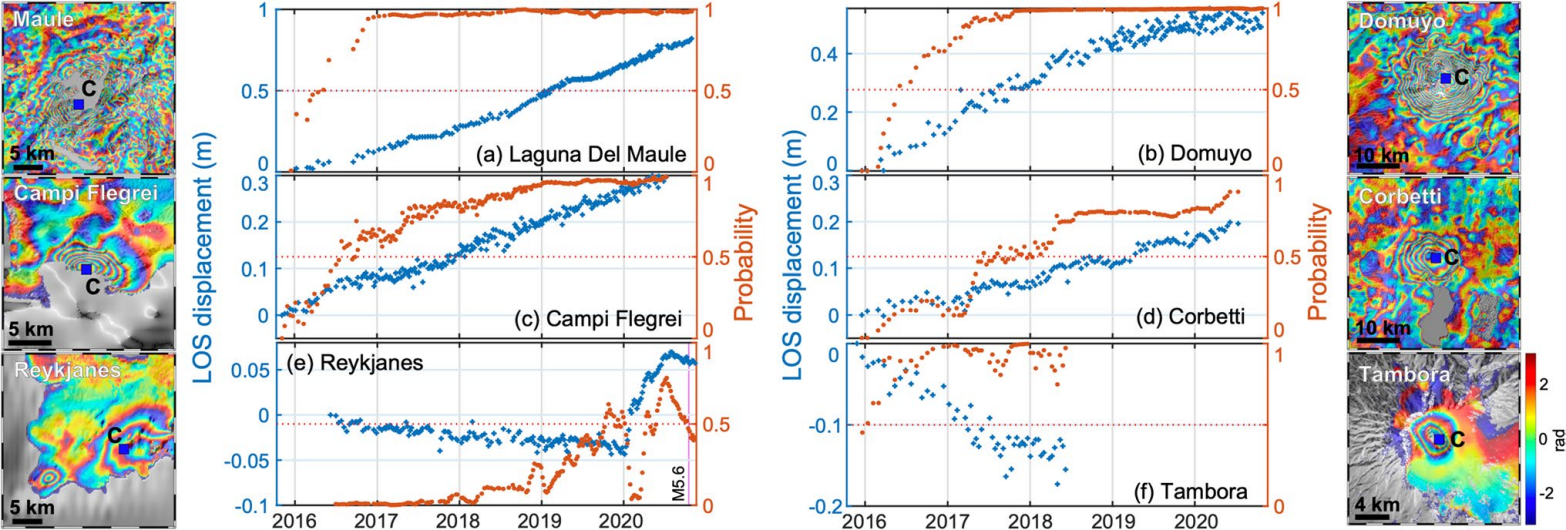
Automated InSAR processing and detection of volcanic unrest. (**a**) Laguna del Maule, Chile; (**b**) Domuyo, Argentina; (**c**) Campi Flegrei, Italy; (**d**) Corbetti, Ethiopia; (**e**) Reykjanes, Iceland; and (**f**) Tambora, Indonesia. Cumulative deformation maps are rewrapped such that each fringe represents 2.8 cm of displacement in the satellite line of sight. The blue square labelled ‘C’ shows the point selected for time-series analysis. Cumulative displacement time series of unwrapped interferograms (blue) with associated probabilities (red)

Many volcanic systems experience deformation at rates of centimetres-per-year for years or decades without resulting in an eruption, especially silicic calderas (Biggs et al. 2014). The CNN can detect these in individual wrapped interferograms if (1) the rate is sufficiently high and (2) there are coherent interferograms with longer time spans. For example, 81 interferograms from Laguna del Maule, Chile, were flagged (Fig. 4, Table 1). Earlier studies have shown uplift at a rate of ~ 20 cm/year between 2007 and 2014 (Le Mével et al. 2016), and our time-series analysis shows a similar rate in 2016–2020, causing a positive flag after just 5 months (Fig. 8a). Similarly, Domuyo, Argentina, began uplifting at a rate of 15 cm/year in 2014 (Lundgren et al. 2020) and was flagged in 63 wrapped interferograms with timespans > 84 days (Fig. 4, Table 1), and after 6 months using the time series approach (Fig. 8b).

Deformation was repeatedly flagged in wrapped interferograms at two volcanic systems with no prior satellite reports of deformation (Ebmeier et al. 2018). The 0.5° × 0.5° tile around the Holocene volcano ‘Reykjanes’ was flagged 52 times (Fig. 4, Table 1). Two areas of deformation are visible: subsidence due to the Reykjanes geothermal power-plant to the west and uplift at Svartsengi in the east (Fig. 8e). The time series from Svartsengi shows slow subsidence from at least mid-2017 consistent with earlier observations which have attributed the deformation to exploitation of the Svartsengi geothermal field (Keiding et al. 2010), with the probability exceeding the detection threshold (*P* > 0.5) on 02-Oct-2019 (Fig. 8e). Uplift at Svartsengi began in early 2020, which briefly reduced the cumulative deformation and hence the probability, before returning to *P* > 0.5 by 19-Apr-2020. Again, this illustrates the challenges associated with using cumulative time series to detect changes in rate. Following the observation period, the unrest led to a dyke intrusion followed by the Fagradalsfjall eruption in March 2021, and can retrospectively be classed as precursory (Flóvenz et al. 2022; Sigmundsson et al. 2022).

Tambora, Indonesia, was the site of world’s largest and deadliest historical eruption in 1815 (Self et al. 1984), but no deformation has been reported since. The Sentinel-1 dataset is sparse over Tambora and only extends to mid-2018, but 19% of the processed interferograms were flagged (Fig. 4, Table 1). The short time-series suggests localised subsidence at a steady rate of 8 los cm/year (Fig. 8f).

#### Non-volcanic signals

Although most volcanoes with a high detection rate experienced deformation associated with volcanic activity, some detections were associated with signals that are non-volcanic in origin. For example, three earthquakes above magnitude 6.4 occurred on Lombok, Indonesia, in July/August 2018, which caused deformation at nearby Rinjani volcano and triggered 52 detections (Fig. 4, Supplementary Fig. 3 in the Online Resource). The CNN flags deformation at Kverkfjöll, Iceland, in 55 interferograms (Fig. 4, Table 1), but the deformation is located along the edge of the ice-cap suggesting it is non-volcanic in origin (Supplementary Fig. 4 in the Online Resource). We identify the signals at Fujisan in Japan and Agung and Lawu in Indonesia as atmospheric in origin and discuss them further in the section on detection performance.

### Occasional detections

We visually inspected the interferograms from volcanoes for which there were occasional (2–25) flags. Many of these are false positives and are easy to filter out during expert review but many are true positives caused by transient or slow deformation. Transient deformation is easy to classify because the flagged interferograms cluster in time. For example, the January 2020 eruption of Taal was associated with lateral magma movement causing large magnitude, complex deformation patterns which were flagged 13 times in individual interferograms, of which 11 spanned January 2020 (Fig. 6b). The time-series shows pre-eruptive deformation of a few centimetres-per-year on the central island, as previously being reported by both ground and space-based sensors (Bato et al. 2020), but the probability remained below the detection threshold, peaking at *P* = 0.4 (Fig. 6f). Another example is Nevados de Casiri in Peru, which was flagged in 11 interferograms, of which 10 spanned July 2020. Visual inspection shows 3 fringes of deformation located ~ 15 km NW of the edifice. The volcano does not have any previous record of Holocene eruption (Global Volcanism Program 2013) or deformation (Ebmeier et al. 2018), but the location and timing of the deformation is consistent with seismic swarm at the end of July 2020 that was reported by the Instituo Geofisico de Peru (Antayhua et al. 2021).

Although some volcanoes experiencing slow rates of deformation were flagged sufficiently often to be classed as ‘persistent detections’ (e.g. Domuyo, Laguna del Maule, Tambora), at others the deformation was too slow to be flagged in many individual interferograms. For example, Alcedo (Galapagos) did not experience any significant eruptive or intrusive activity during the observation window, but slow subsidence (< 3.5 los cm/year) within the caldera was sufficient to cause 12 detections in the short-term interferograms (Table 1) and for the time series to exceed *P* = 0.5 after 6.5 months (Fig. 5e). Other examples such as Campi Flegrei, Italy (8.5 cm/year since 2017) (Anantrasirichai et al. 2019a), and Corbetti, Ethiopia (6–7 cm/year) (Albino and Biggs 2021), were only flagged once or twice in individual interferograms, but deformation can easily be detected using a cumulative time series approach (Fig. 8c,d).

## Detection performance

In this study, we use an expanded dataset of ~ 600,000 images for testing, which allows us to consider the performance of the machine learning algorithm under a wider range of atmospheric conditions, signal characteristics and land cover types than was previously possible. In this section, we consider the effects that these factors have on detection. First, we calculate a formal detection threshold using the time-series results, and then we explore the effects that atmospheric conditions, coherence and deformation characteristics have on detectability. We use contrasting regions as case studies: we consider atmospheric effects in Asia, coherence in the Chilean Andes and signal characteristics in Afar, Ethiopia.

### Detection threshold

**Fig. 9:**
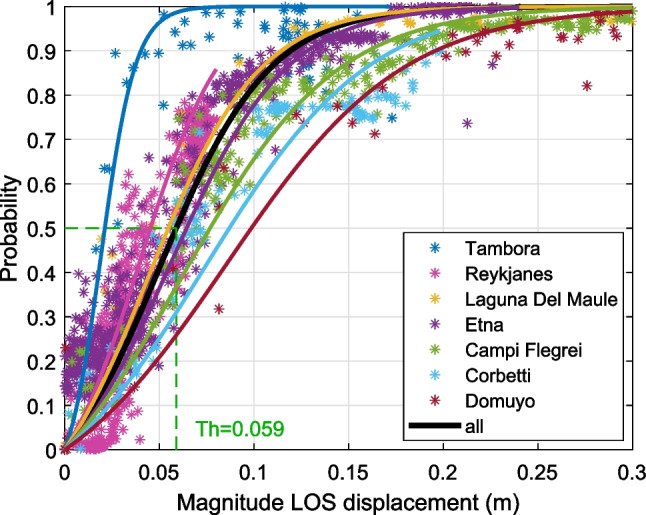
Detection threshold of the CNN calculated from the time series. Tambora = 2.1 cm, Reykjanes = 4.5 cm, Laguna Del Maule = 5.5 cm; Etna = 6.3 cm; Campi Flegrei 7.7; Corbetti = 8.7 cm and Domuyo = 9.9 cm

We use the time series results to estimate the detection threshold of the CNN. We plot the output probability, *P*, plotted against maximum displacement for each step of the time series (Fig. 9) and fit a sigmoidal curve, defined as *f*(*x*) = (1 + *e*^−*a*(*x*−*b*)^) following Anantrasirichai et al. (2019a). The transition between *P*∼0 (undetectable) and *P*∼1 (detectable) occurs over a narrow range and corresponds to the minimum value of displacement that the CNN can identify. The detection threshold for the whole dataset is 5.9 cm, equivalent to a rate of 1.2 cm/year over the 5-year study period. However, it is important to note that this estimate is based on time-series generated for volcanoes where deformation has been detected, and the thresholds might be significantly higher elsewhere.

There is considerable variability between volcanoes with individual values ranging from 2.1 cm (0.4 cm/year) at Tambora, Indonesia, to 9.9 cm (2.0 cm/year) at Domuyo, Argentina. In general, detection thresholds are < 5 cm for low relief volcanoes such as Reykjanes and Tambora but are > 5 cm for high relief volcanoes such as Laguna del Maule, Etna and Domuyo. These values are consistent with similar tests conducted on synthetic data by Anantrasirichai et al. (2019a). Volcanoes where part of the deformation signal is obscured by vegetation, such as Corbetti, or water cover, such as Campi Flegrei, also tend to have high detection thresholds (> 7.5 cm).

### Atmospheric artefacts

**Fig. 10:**
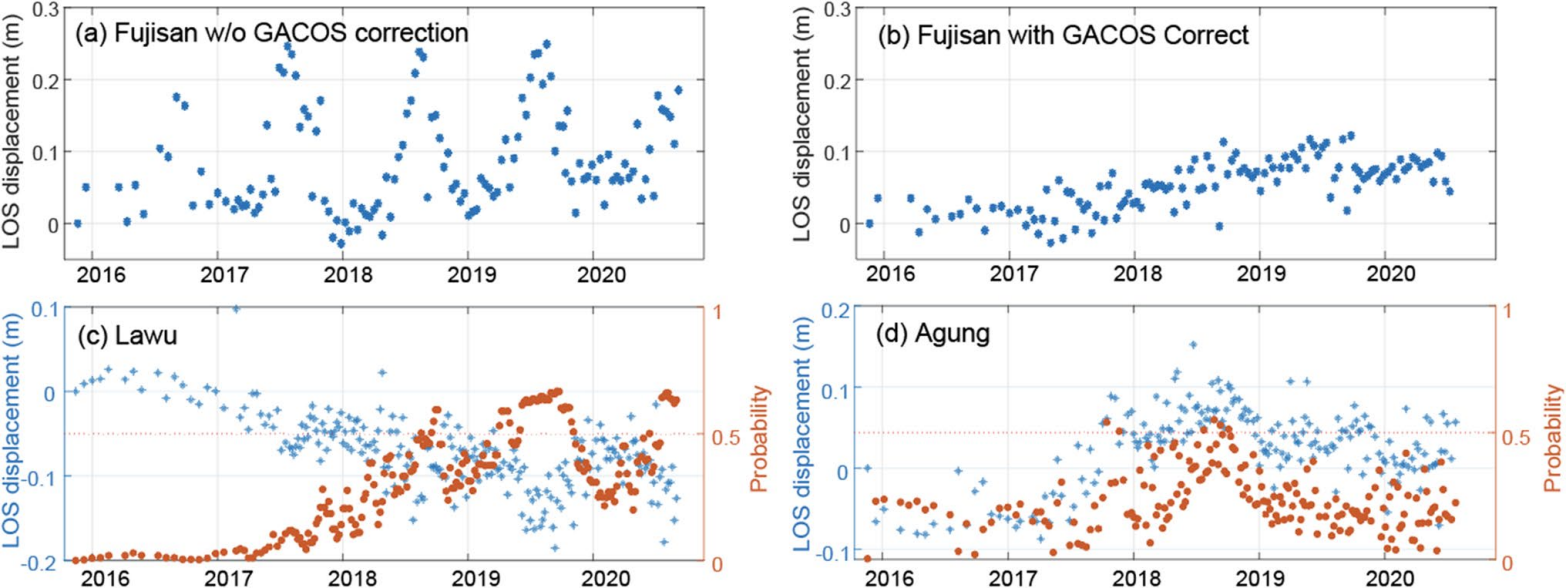
Automated processing and machine learning results for Fujisan in Japan and Agung and Lawu in Indonesia. (**a**) Fujian timeseries without GACOS correction which is dominated by a seasonal signal. (**b**) Fujian timeseries using GACOS correction to remove atmospheric contributions, (**c**) timeseries show cumulative displacement (blue) and probability (red) for selected point at Lawu, Indonesia and (**d**) timeseries show cumulative displacement (blue) and probability (red) for selected point at Agung, Indonesia

Atmospheric effects are a major challenge for InSAR, especially at high-relief volcanoes (Albino et al. 2020; Beauducel et al. 2000; Bekaert et al. 2015). Although the model of Anantrasirichai et al. (2019b) was trained using synthetic data from global atmospheric models to distinguish between deformation and atmospheric artefacts, that study identified false positives due to atmospheric artefacts in 53 of the ~ 30,000 interferograms tested. In this expanded study, expert review suggests that 3 of the 16 volcanoes with persistent detections can be attributed to atmospheric artefacts, including 165 flags (the second highest) at Fujisan, Japan (Fig. 4, Table 1). The signal at Fujisan is seasonal (Fig. 10a), and similar signals reported in large scale Sentinel-1 studies of Japan and have been attributed to tropospheric water vapour variations and snow loading (Morishita et al. 2020). The other persistent detections attributed to atmospheric artefacts are at Agung and Lawu, Indonesia, which were flagged 31 times each by the algorithm, but only 1–2 times with *P* > 0.9. In both cases, the time-series are very noisy, and the detection probability rarely exceeds 0.5 (Fig. 10c,d). Atmospheric artefacts are particularly common at tropical island volcanoes and have led to misinterpretation of InSAR signals in the past (Rémy et al. 2015; Yip et al. 2019). At Agung, this atmospheric noise actually masks the deformation which preceded the 2017 eruption (Albino et al. 2019) and demonstrates the importance of atmospheric corrections.

Anantrasirichai et al. (2019b) found that applying a correction from a global atmospheric model (Yu et al. 2018) reduced the probability for false positives below the threshold of 0.5. In the case of Fujisan, the GACOS correction reduces the number of detections to from 165 to 64, but does not completely remove them (Fig. 10b). The residual rate of 1.5 los cm/year is significantly higher than the ± 2.4 mm/year misfit (1 s.d.) between GNSS stations and InSAR velocities on the Kanto plain to the north but consistent with misfits to levelling data of around 1 cm/year for individual points within Niigata City (Morishita et al. 2020). Discrepancies in residual deformation between overlapping tracks suggest this is an artefact associated with the extreme topography, seasonal snow cover and poor coherence at Fujisan.

### Interferometric coherence

**Fig. 11:**
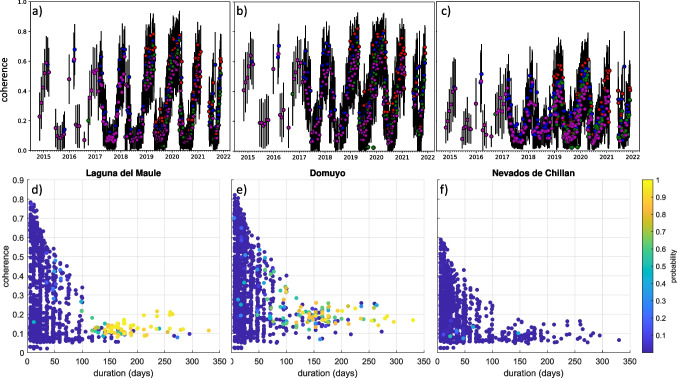
The influence of interferometric coherence on signal detectability illustrated using 3 deforming volcanoes in the Andes: (**a**, **d**) Laguna del Maule, (**b**, **e**) Domuyo and (**c**, **f**) Nevados de Chillan. (**a**–**c**) Seasonal variations in coherence. Red, 6-day interferograms; blue, 12-day interferograms; green, 18-day interferograms; magenta, 24-day inteferograms. (**d**–**f**) CNN detection probability as a function of interferogram coherence and length

Next, we consider the case of the Chilean Andes where high relief and seasonal snow cover represent challenging conditions for automated systems. Furthermore, this is an environment that was not well-represented in the original training or testing datasets of Anantrasirichai et al. (2018, 2019b). We focus on 3 rapidly uplifting volcanoes with similar deformation rates and footprints: Laguna del Maule (2162 m) was uplifting at a rate of 20 cm/year, Domuyo (4702 m) was uplifting at a rate of 15 cm/year and Nevados de Chillan (3180 m) began uplifting at a rate of ~ 10–12 cm/year in July 2019 (Astort et al. 2022). Each of these volcanoes is covered by ~ 1400 interferograms, but the number of detections is very different: Laguna del Maule was flagged 61 times, Domuyo 63 times and Nevados de Chillan just once. Figure 11a–c shows that the coherence at all 3 volcanoes is highly seasonal, but while coherence at Laguna del Maule and Domuyo reaches values of ~ 0.8 for short, summer interferograms, the maximum at Chillan is 0.6. For detecting slow deformation, long duration interferograms are especially important, and the majority of positive detections at Laguna del Maule and Domuyo come from interferograms with durations of > 100 days (Fig. 11d–e). This corresponds to deformation of 4–5 cm, consistent with the detection threshold estimated by Anantrasirichai et al. (2019a) based on synthetic tests. At Nevados de Chillan, these longer interferograms are less coherent, and the detection probability remains very low (Fig. 10f).

Although detectability does not depend on coherence alone, we find that the mean coherence of the interferograms, which is automatically calculated for each track, is a useful indicator of areas where the algorithm may not perform well. At Laguna del Maule and Domuyo, the mean coherence ranges from 0.24 to 0.33. In contrast, at Nevados de Chillan the mean coherence is just 0.13–0.20. Similarly, several other notable volcanoes with good coverage but low mean coherence were not flagged, such as Yellowstone (0.12) and Masaya (0.08–0.11). In both these cases, tailored processing would be required for the signal to be detectable. In summary, we demonstrate that the CNN can detect slow deformation in individual interferograms if (1) the rate is sufficiently fast, (2) there are interferograms with long time spans and (c) those interferograms are sufficiently coherent.

### Signal characteristics 

In this section, we focus on the Afar region of Ethiopia where Albino and Biggs (2021) and Moore et al. (2021) have carried out detailed analyses of the Sentinel-1 dataset. The testing dataset contains 30,275 interferograms covering the 31 volcanoes in Afar (Table 2), of which 6 have known deformation: Alu-Dalafilla, Dabbahu-Hararo, Dallol, Erte Ale, Gada Ale and Nabro (Albino and Biggs 2021). The mean coherence is > 0.34 (Table 2) and the volcanoes are mostly low relief such that atmospheric artefacts are negligible. Thus, this is a good region to test how detectability depends on the characteristics of the signal.

**Table 2:** Systematic analysis of the 31 volcanoes in Afar. Volcanoes with deformation reported by Albino and Biggs (2021) are shown in bold to provide ground truth. *TP*, true positive; *TN*, true negative; *FP*, false positive; * denotes examples where the detection was associated with another signal as described in the notes column. For TP, the rate of deformation is given. The total number of false positives is 241, or 0.8% of the 30,275 interferograms in the Afar dataset

Volcano	Mean coherence	Number images	Number flags	Expert review	Notes
**Erta Ale**	0.75	798	42	TP	± 200/year
Ale Bagu	0.72	808	41	FP*	Erte Ale
Asavyo	0.64	992	41	FP*	Nabro
Hayli Gubbi	0.73	810	37	FP*	Erte Ale
Bora Ale	0.73	798	27	FP*	Erte Ale
Dubbi	0.67	1039	19	FP	
Alayta	0.78	798	17	FP	
**Alu-Dalafilla**	0.71	798	17	FP*	Erte Ale
Ardoukoba	0.59	1674	17	FP	
Mat Ala	0.7	798	15	FP	
Assab	0.8	783	12	FP*	Lake
Ayelu	0.34	1006	9	FP	Atmos
**Dabbahu**	0.6	1178	9	TP	3.9 cm/year
Tat Ali	0.7	810	9	FP*	Lake
**Nabro**	0.63	1299	6	TP	3.1 cm/year
Borawli	0.74	2213	5	FP	
**Dallol**	0.54	1026	4	FP	− 3.3 cm/year
Dama Ali	0.55	782	4	FP	
Dabbayra	0.44	859	3	FP	
Ma Alalta	0.57	798	3	FP	
Mousa Alli	0.67	783	3	FP	
Adwa	0.37	776	2	FP*	Atmos
Alid	0.63	638	2	FP	
**Gada Ale**	0.56	1122	2	TP	− 1.9 cm/year
Gufa	0.84	783	2	FP	
Gabillema	0.67	782	1	TN	
Manda-Inakir	0.57	1251	1	TN	
Yangudi	0.32	1151	1	TN	
Groppo	0.35	415	0	TN	
Manda Gargori	0.38	1415	0	TN	
Manda Hararo	0.6	1092	0		

At Erte Ale, a dyke intrusion caused short-term deformation rates in excess of 200 cm/year triggering 42 flags; Dabbahu, Nabro and Dallol in Ethiopia were all deforming at steady rates of 3–4 cm/year and were flagged 4–10 times and the small, slow subsidence signal at Gade Ale (1.9 cm/year) was only flagged twice. Although Alu-Dalafilla was flagged 17 times, this was because of its proximity to the large signal at Erte Ale, and the slow deformation signal (1.2 − 1.6 cm/year) reported by Albino and Biggs (2021) was below the detection threshold. The algorithm initially identified a further 25 volcanoes that were not picked out by Albino and Biggs (2021), including 4 volcanoes with more than 25 flags each (Table 2). Many of these are false positives associated with closely spaced volcanoes and are easy to filter out during expert review: for example, Ale Bagu, Hayli Gubbi and Bora Ale were flagged because the signal at Erte Ale appears within their frame, and similarly Asavyo was flagged due to the nearby signal at Nabro. The remaining 122 interferograms represent a false positive rate of 0.3–2.1% per volcano, and just 0.4% for the Afar region as a whole. Some of these false positives are caused by atmospheric artefacts (Adwa, Ayelu), while others are associated with the edge of lakes (e.g. Borawli, Mat Ala, Tat Ali).

## Discussion and conclusions

Satellite systems are ideally suited for global environmental monitoring, and several pilot studies have shown the potential for using machine learning approaches for characterising and detecting volcanic deformation (Anantrasirichai et al. 2019a, 2019b; Bountos et al. 2022, 2021; Gaddes et al. 2019; Sun et al. 2020). This study is the first to demonstrate the powerful combination of automatically processed satellite data and machine learning on a large global dataset: we process and analyse more than half-a-million images acquired by Sentinel-1 in a 5-year period and covering > 1000 volcanoes. This enables us to test the model on a much wider range of land cover, atmospheric conditions and signal characteristics than previously possible.

Of the 16 volcanoes flagged most frequently by the convolutional neural network: five were associated with significant eruptions (Sierra Negra, Kilauea, Etna and Erta Ale, Fernandina) and six examples with non-eruptive unrest (Cerro Azul, Wolf, Domuyo, Laguna del Maule, Reykjanes and Tambora). The deformation on the Reykjanes Peninsula is retrospectively considered a precursor to the 2021 Fagradalsfjall eruption, which occurred after the end of our observation period. Among these are several new observations that have not been previously published: (1) a completely new deformation signal at Tambora, Indonesia; (2) a change in rate of deformation at Wolf, Galapagos, from 3 cm/year prior to the 2015 eruption (Xu et al. 2016) to 6 cm/year during our study period of 2016–2020; (3) a reversal in deformation at Fernandina, Galapagos, in January 2020, coinciding with a minor effusive eruption reported by Vasconez et al. (2018). Similar minor effusive eruptions in September 2017 and June 2018 may have caused a decrease in rate of uplift. We also identify deformation near Nevados de Casiri in Peru associated with a seismic swarm reported by the Institute Geofisico de Peru (Antayhua et al. 2021). Although designed for detecting volcanic deformation, the machine learning algorithm is also capable of identifying non-volcanic signals, such as those at Kverkfjöll, Fujisan and Rinjani, and has considerable potential in other applications (Anantrasirichai et al. 2020).

By testing machine learning algorithms on a more representative global dataset, we are able to make recommendations for the development of global volcano monitoring systems. We find that the performance of the machine learning algorithm is primarily limited by the quality of the available data, with poor coherence and slow signals being particularly challenging. We show that individual wrapped interferograms are best suited for detecting eruptions and intrusions, which are characterised by sudden, large deformation signals. Slow deformation associated with unrest can also be detected in individual interferograms if they retain coherence over a sufficiently long timespan, but the use of timeseries is more reliable and provides insight into the temporal behaviour of the signal. However, the use of cumulative time series can delay detection if the new signal reverses sign, as shown at Kilauea and Svartsengi, and machine learning methods designed to detect changes in rate and/or pattern are more suited to these cases (Gaddes et al. 2019).

For machine learning algorithms to become fully integrated into automated processing systems, some adaptations to the current processing strategies are needed. Specifically, we show that (1) long-duration interferograms or timeseries of unwrapped images are needed to detect slow deformation associated with unrest; (2) tailored processing strategies are needed in regions with dense vegetation and/or snow cover, where automated processing systems may produce incoherent interferograms; and (3) atmospheric corrections using global weather models reduce the total number of false positives, but atmospheric artefacts can still cause false positives or mask real deformation. Many of these recommendations are already been implemented in the LiCSAR system to avoid issues related to cumulated unwrapping errors and fading phase bias (Maghsoudi et al. 2022). For new acquisitions, interferograms connecting the spring and autumn seasons with year-long durations are now automatically generated (Lazecky et al. 2021), and these will be particularly important for building a more complete catalogue of slowly deforming volcanoes.

Although the analysis presented here was conducted retrospectively, the underlying motivation is to design a real-time monitoring and alert system. To this end, the machine learning algorithms described here have been adapted to run on the open-access, web-based COMET Volcano Portal (https://comet.nerc.ac.uk/comet-volcano-portal/). The portal displays a range of LiCSAR products, including interferograms and time series with interactive tools designed to allow observatory volcanologists to (1) search quickly through processed imagery for deformation and (2) to make a critical assessment of whether any apparent signals are likely to be noise or true displacements (Ebmeier et al. 2013; Biggs et al. 2021). The machine learning algorithm of Anantrasirichai et al. (2019b) runs automatically and provides users with a tool for quickly finding major deformation events in a time series. Further developments in both automated processing and machine learning tools should improve the reliability and timeliness of this service further.

## Open research

The InSAR data are available at http://comet.nerc.ac.uk/COMET-LiCS-portal/.

Machine Learning Codes are available at: https://doi.org/10.5281/zenodo.5550815.

The machine learning outputs (filenames and probabilities) are included as a supplementary datafile and is also available at https://doi.org/10.5281/zenodo.7199356. Datacubes for the 20 volcanoes studied in depth are available at https://doi.org/10.5281/zenodo.7213407.

## Supplementary Information

Below is the link to the electronic supplementary material.

Supplementary file1 (PDF 3089 KB)
