# Supplementary material for: Large-scale demonstration of machine learning for the detection of volcanic deformation in Sentinel-1 satellite imagery
Source: Bull Volcanol. 2022 Nov 3;84(12):100. doi: 10.1007/s00445-022-01608-x (PMC9633547; doi:10.1007/s00445-022-01608-x)
Supplement: Supplementary file 1 — Supplementary file1 (PDF 3089 KB) [file 445_2022_1608_MOESM1_ESM.pdf]

Supplementary Information

Large-scale demonstration of machine learning for the detection of volcanic deformation in Sentinel-1 satellite imagery

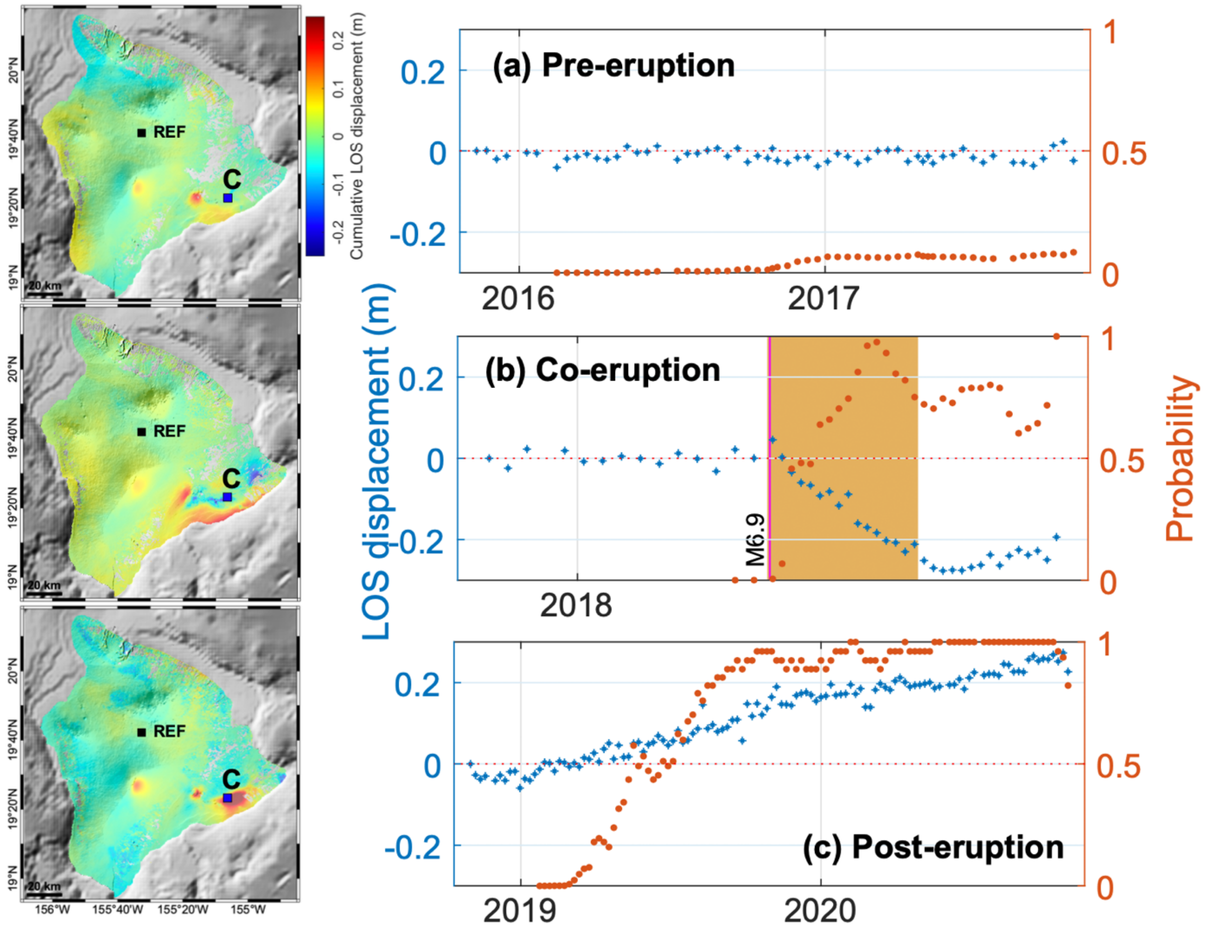

Supplementary Figure 1. Automated processing and machine learning results for Kilauea, Hawaii. Left: Cumulative displacement maps (unwrapped) for the Big Island of Hawaii for November 2015-November 2020. a-c) timeseries showing cumulative displacement at point C (blue) and probability (red) for a) pre-eruptive, b) co-eruptive and c) post-eruptive time-periods. The cumulative displacement is reset to zero at the start of each time period. Eruptions are shown in orange and notable earthquakes in pink (with magnitude).

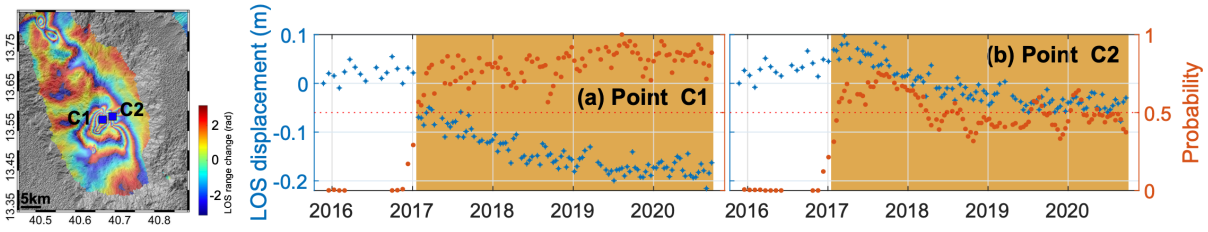

Supplementary Figure 2. Automated processing and machine learning results for Erta Ale, Ethiopia. Cumulative displacement map (wrapped modulo 2.8 cm) for November 2015-

November 2020. a-b) timeseries showing cumulative displacement (blue) and probability (red) for selected points. a) Point C1 is close to the summit and deformation is dominated by the dyke intrusion. b) Point C2 shows gradual subsidence of the lava flows. Intr

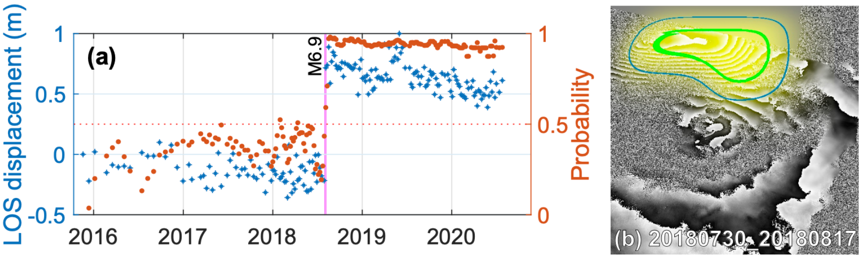

Supplementary Figure 3. Automated processing and machine learning results for Rinjani, Indonesia. a) timeseries showing cumulative displacement (blue) and probability (red) for selected point. Pink line denotes timing of the M6.9 earthquake. b) Machine learning output superimposed on an interoferogram spanning the earthquake, showing that the fringes detected as associated with the earthquake, not the volcanic edifice.

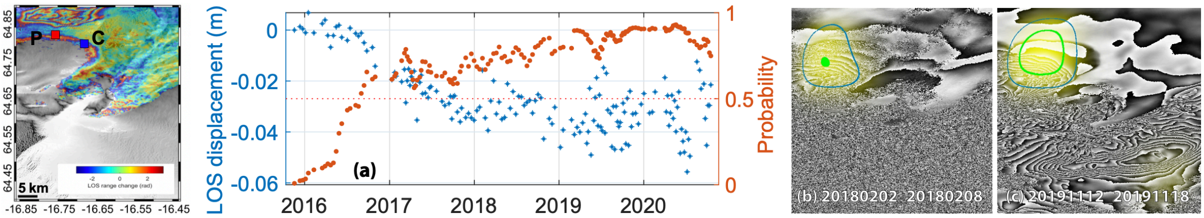

Supplementary Figure 4. Automated processing and machine learning results for Kverkfjöll, Iceland. a) timeseries showing cumulative displacement (blue) and probability (red) for selected point b,c) example machine learning outputs superimposed on wrapped, greyscale interoferograms. The cumulative deformation image (a) shows deformation following Bárðarbunga-Holuhraun (post-rifting or cooling of the lava field), but this is not flagged in the individual interferograms (b,c). Instead, the CNN detects fringes located on the ice sheet itself which are non-volcanic in origin.
